# Children’s exposure to unhealthy food advertising on Philippine television: content analysis of marketing strategies and temporal patterns

**DOI:** 10.1080/16549716.2024.2427445

**Published:** 2024-11-21

**Authors:** Elaine Q. Borazon, Ma. Rica Magracia, Gild Rick Ong, Bridget Kelly Gillott, Sally Mackay, Boyd Swinburn, Tilakavati Karupaiah

**Affiliations:** a International Graduate Program of Education and Human Development, National Sun Yat-sen University, Kaohsiung, Taiwan; b School of BioSciences, Faculty of Health & Medical Sciences, Taylor’s University Lakeside Campus, Selangor, Malaysia; c Early Start, School of Health and Society, University of Wollongong, Wollongong, Australia; d Faculty of Medical and Health Sciences, Epidemiology and Biostatistics, University of Auckland, Auckland, New Zealand; e School of Population Health, University of Auckland, Auckland, New Zealand

**Keywords:** Children, food, marketing, persuasive techniques, television, advertisements

## Abstract

**Background:**

This study conducted an exploratory content analysis of TV food advertisements on the top three most popular channels for Filipino children aged two to 17 during school and non-school days.

**Methods:**

Data were collected by manually recording of aired advertisements from 16 non-school days (July to September 2020) and 16 school days (January to April 2021). Descriptive and inferential statistical analyses were used to assess children’s rates of exposure to food advertisements (mean ± SD of advertisements aired per channel per hour), the healthiness of promoted foods (as permitted (healthier) or not permitted (unhealthy) according to nutrient profiling models from the World Health Organization), and persuasive techniques used in food advertisements, including promotional characters and premium offers.

**Results:**

The results show that the rates of exposure to food advertisements were higher during school days (14.6 ± 14.8) than on non-school days (11.9 ± 12.0) (*p* < 0.01). Both periods yield a similarly higher proportion of non-permitted food advertisements (e.g. 9.3 ± 9.7 ads/channel/hour for school days and 8.3 ± 8.5 ads/channel/hour for non-school days) than permitted ones. More non-permitted food advertisements during children’s peak viewing times were observed than non-peak viewing times (e.g. 11.8 ± 10. vs. 8.3 ± 9.2 ads/channel/hour for school days). Non-permitted food advertisements employed persuasive techniques more frequently, accounting for 64–91% of all food ads during peak viewing times.

**Conclusion:**

Children are exposed to a large volume of television advertisements for foods that should not be permitted to be marketed to children based on authoritative nutrient criteria.

## Paper Context

- **Main findings:** Filipino children experienced a significantly higher rate of food advertisements per hour during school days compared to non-school days, with non-permitted food advertisements being more frequent than permitted ones, especially during peak viewing times.
- **Added knowledge:** A large percentage of food advertisements were for non-permitted food products, with common persuasive techniques including the use of images of children, celebrities, and various premium strategies, such as ‘20% extra’ and price discounts, which target both children and their parents.
- **Global health impact for policy and action:** The findings support the need for stringent regulations on TV food marketing to children, including the development of clear standards for permitted versus non-permitted food advertisements, redefining children’s peak viewing times for more effective monitoring, and extending regulations to other media platforms to curb the influence of unhealthy food marketing on children’s dietary choices and overall health.

## Background

Each year, around 41 million deaths worldwide are attributed to non-communicable diseases (NCDs) [1]. Low- and middle-income countries (LMICs) have a higher prevalence of NCD-related deaths than high-income countries, accounting for 77% of the global cases [1]. The Philippines, also an LMIC, is not immune from this trend with 70% of all mortality attributed to NCDs [2]. One of the leading risk factors contributing to the rising cases of NCDs in LMICs is malnutrition (WHO, 2020a). A recent report from the World Health Organization (WHO) stated that 39 million children less than 5 years old were classified as either overweight or obese in 2020 (WHO, 2021). Although undernutrition has been a well-recognized public health issue in the Philippines for decades [3], an emerging childhood issue is obesity. This double burden of malnutrition is common in LMICs undergoing a nutrition transition to a Westernized food supply, comprising ultra-processed foods and sugar-sweetened beverages, and displacing traditional diets [4]. In the Philippines, obesity rates for children were observed to double in three years, from 7.6% in 2018 to 14% in 2021, based on a nationwide nutrition survey [3]. The rapid escalation of numbers needs to be addressed urgently, given that overweight or obese children are known to have higher chances of maintaining their excess weight until adulthood which increases their premature risk of developing NCDs [5].

The increasing problem of overweight and obesity in children can be linked to factors such as ‘poor diets, failing food systems, and lack of physical activity’ [6]. Children situated in LMICs are more likely to consume unhealthy food with high levels of sugar and fat [7]. One of the key drivers of unhealthy food consumption is environmental exposure to the marketing of unhealthy products, which can influence the dietary behaviors of both children and adults [8–10]. Several studies [11–13] show that advertised foods are usually unhealthy, and children are more influenced by advertising practices in terms of purchasing requests, food choices, and consumption than adults. Content analysis of advertisements in TV channels in various countries has revealed that foods are some of the most advertised products and most food advertisements promote unhealthy foods such as sugary snacks and drinks and fast food [14–18]. The excessive exposure of children to advertisements promoting unhealthy food consumption necessitates regulatory policies to limit their frequency and impact on children [19].

Despite the rise of the internet and online media, television remains one of the major platforms for media consumption in the Philippines [20]. Television has been described as the ‘center’ of Filipino homes [21] and has become a family source of entertainment [22]. Around 21.7 million or 80% of the households have televisions and 92.9 million individuals regularly watch television [23]. The extreme popularity of television has stemmed from its extensive coverage in the country, especially in rural areas where digital infrastructure is not fully developed [24]. Consequently, advertisements shown on television have a wide reach and serve as a main vehicle for companies to exert influence over consumers [25]. Studies have shown that television audiences are often imagined as families and television consumption is primarily done at home. As such, televisual representation of families is commonly observed in family dramas, soap operas, and even in advertising [26].

The relevance of television in the Philippine household has resulted in high television exposure among family members. The 2019 Function Literacy, Education, and Mass Media Survey (FLEMMS) revealed that the population is more exposed to television daily (66%) compared to other mass media, including radio, print, and online media [27]. Recent statistics show that Filipinos spend an average of 3.10 hours per day engaging with free-to-air television, which is still close to the number of hours spent on social media at 3.43 hours daily [28]. Children also watch television for extended periods each day, generally 3 hours per day compared to the recommended maximum of 2 hours per day [21]. Children tend to watch in high numbers during ‘primetime’ (5 p.m. to 10 p.m.) and noontime variety shows [21], which are not intended for them and may require parental guidance.

Children’s exposure to television varies during school and non-school days. Numerous studies show that adolescents have more screen time exposure during non-school days compared to school days [29–31]. This was also observed in the Philippines, where morning and afternoon timeslots during weekdays have a low number of children viewers attributed to their school schedules [21]. In contrast, weekends provide more options for children as they get to watch television at any time of the day [21]. Excessive exposure to screen time may negatively impact nutrition outcomes, particularly overweight and obesity [32] and unhealthy diet [33]. Adverse impacts of television viewing were primarily caused by prime-time commercial ads promoting unhealthy dietary practices [34,35]. Notably, only 4% of food ads shown during children’s viewing time are found to be healthy [36].

There are limited regulations related to TV food marketing to children in the Philippines. The National Council for Children’s Television (NCCT) serves as the primary agency dedicated to fostering a child-friendly media environment for Filipino children [37]. In line with their mandates, NCCT’s Memorandum Circular No. 2019–01 enumerates criteria for children’s television programs and child-friendly television programs which includes a section for monitoring the nutritional aspects in Section 2a [5]: ‘Nutrition – Portrayals, or references to food and beverages, do not promote or condone unhealthy eating’ [38]. However, there is a lack of a definitive standard on what food products are to be considered healthy or not. In addition, leading food manufacturers have committed to responsible marketing for children below 12 years old through the 2014 Philippine Pledge on Responsible Advertising to Children. The pledge stipulates that only food products that meet the nutritional criteria and dietary guidelines for children under 12 years old can be advertised [39]. In other countries, adherence to voluntary codes has not sufficiently reduced TV advertising of unhealthy food and beverages, and therefore this policy approach has proven to be ineffective [40,41]. Mandatory government policy approaches aligned with WHO recommendations are found to be more effective in limiting food marketing to children than self-regulations [42].

As television remains a major source of media in Filipino households, it is important to explore the current content of TV advertisements and children’s potential exposure. This study aims to 1) assess the level of children’s exposure to TV food advertisements during school and non-school days; 2) examine the healthiness of TV food advertisements during school and non-school days using WHO regional nutrient profiling models; and 3) identify the persuasive marketing strategies used in TV food advertisements during school and non-school days. Understanding the current landscape of TV food advertisements will allow for better policy design, consumer protection, educational interventions, and industry accountability to safeguard children’s health.

## Methods

### Study sample

The top three TV channels were determined based on viewership data from May 2019 to August 2020 provided by the Nielsen Company (Philippines). The top five 1-hour peak viewing periods were also selected based on the viewing times with the highest child audiences (aged 2–17) during weekend days (11 a.m.–1 p.m. and 7 p.m.–10 p.m.) and weekdays (10 a.m.–12 p.m. and 7–10 p.m.). According to the Nielsen Report [43], Global Media Arts (GMA), GMA News TV (GNTV), and TV5 were the three most-watched free-to-air channels by children.

### Period of data collection

The data collection was conducted during both non-school and school days to capture how children’s television viewing patterns and exposure to food advertisements vary based on their daily routines. School days were defined as days when children had to attend school, either in-person or through remote learning classes, whereas non-school days were school breaks for children during the COVID-19 quarantine period. Children’s TV viewing opportunities vary between these periods and might be exposed to targeted food advertising during specific windows (before and after school hours) during school days, while non-school days offer more flexible viewing times. Eight weekdays and eight weekend days were selected within the three months. Data were captured for 16 non-school days between July and September 2020 and for 16 school days between January and April 2021. This sampling strategy enabled us to systematically examine how advertising strategies adapt to reach children during different daily activity patterns in the Philippine context.

### Recording process

Data collection was conducted through manual recording of live TV including advertisements. The TV recordings were viewed, and advertisements were classified according to predetermined criteria. The coding sheet design included basic information such as channel, date, airtime, and name of the program. ‘Programs for children’ were identified as cartoons, movies, series, or other shows intended for children’s viewing. A total of 864 recorded hours of television broadcast were obtained during school and non-school days.

### Measures

The study adopted the 2017 INFORMAS (International Network for Food and Obesity/Non-communicable Diseases Research, Monitoring and Action Support) protocol for monitoring food promotions on television [44]. The module aims to develop a standardized monitoring system for measuring the extent and nature of TV food and non-alcoholic drink promotions for children that can be used for cross-country comparisons over time [44]. Food advertisements largely include foods and beverages, nutritional supplements, baby food, follow-up formula, and kitchen ingredients, as well as food advertisements without food products (food brands only). Food advertisements were classified as ‘permitted’ or ‘non-permitted’ to be marketed based on the WHO Western Pacific Regional Office (WPRO) and Southeast Asian Regional Office (SEARO) nutrient profile models. The two nutrient profile models were developed by WHO to aid in the classification of foods in policies to protect children from the marketing of unhealthy and non-alcoholic beverages [45,46]. Food advertisements that did not fit within the food categories in the two models are classified as ‘not applicable,’ ‘no nutrient information panel,’ or ‘brand only’. Food advertisements that were ‘not applicable’ include baby food, baby and follow-up formulae, vitamins/supplements, and kitchen ingredients. Kitchen ingredients (see supplementary Table B for full list) were determined and separated into another category because these products (e.g. herbs and spices, seasonings, cooking sauces) were intended for cooking and hence do not intentionally target children. The team decided to only include foods and beverage products that may have a potential impact on children’s purchasing requests and consumption behaviors after the food advertisement exposures.

We also identified food advertisements that did not have sufficient nutritional information (‘no nutrient information panel’) to determine their healthfulness, such as fast-food items. Nutrition information was estimated for these foods, where possible, using the Philippine Food Composition Tables Online Database (PhilFCT®) [47] and Energy and Nutrient Composition of Food [48]. ‘Brand Only’ advertisements were those promoting a company brand only without food products. Furthermore, the use of persuasive strategies in food advertisements was assessed, including promotional characters and premium offers (e.g. price discounts, giveaways, game and application downloads). Before the analysis, three coders independently coded a random sample of television data (*n* = 1 hour) to assess the correlation between the results and identify any coding discrepancies. The inter-coder agreement among the coders was required to be at least 90% to ensure reliability.

### Statistical analysis

The coded datasets were aggregated by the sum of advertisements aired per 1-hour timeslot for each of the three channels. Frequency analyses on the non-aggregated datasets were conducted to compare trends in food advertisements by WPRO and SEARO food categories and by persuasive techniques used. Statistical analyses were conducted using SPSS Statistics® Version 27 to compare the rates of advertising for permitted and non-permitted food advertisements during school and non-school days. The level of exposure of children to food advertisements was measured using the mean and standard deviation (mean ± SD) of advertisements aired per channel per hour. Mann-Whitney U test was used to compare the differences between school and non-school groups and non-permitted food ads during peak and non-peak viewing times of children. Wilcoxon signed-ranks test was used to compare the rates of permitted and non-permitted food ads within groups. Additional weights for the data set were calculated based on the inverse probability of selection to account for the unequal chances of selection during weekends and weekdays.

## Results

### Level of exposure to food advertisements

A total of 864 hours of TV were captured, half for school days and a half for non-school days to capture distinct patterns in children’s television viewing behaviors and exposure to food marketing across different temporal contexts, as children have structured viewing times that advertisers may specifically target. The overall number of advertisements during school days was 40,094, of which 13,492 were food advertisements. For non-school days 36,224 advertisements were recorded and 11,119 were food advertisements. The rates of all advertisements (ads/channel/hour) observed during school and non-school days are shown in Table 1. There were significantly higher rates of advertisements shown during school days (45.5 ± 24.8, 95% CI 44.4–46.5) compared to non-school days (41.1 ± 26.2, 95% CI 39.9–42.2) of children (*p* < 0.001).

**Table 1.** Rate of all advertisements, food advertisements, and non-food advertisements (ads/channel/hour) during school and non-school days (α = 0.05).

Indicator (ads/channel/hour)	School days	Non-school days	*p*^a^
All Ads Mean ± SD Median (IQR) Min Max 95% CI N	45.5 ± 24.8 44 (29, 64) 0 111 44.4 – 46.5 40,094	41.1 ± 26.2 35 (20, 61) 0 126 39.9 – 42.2 36,224	<0.001
Food Ads Mean ± SD Median (IQR) Min Max 95% CI N	14.6 ± 14.8 9 (2, 25) 0 62 13.9 – 15.2 13,492	11.9 ± 12.0 8 (2, 19) 0 57 11.4 – 12.5 11,119	0.002
Non-Food Ads Mean ± SD Median (IQR) Min Max 95% CI N	30.9 ± 15.4 32 (20, 41) 0 72 30.3 – 31.6 26,602	29.1 ± 17.0 26 (15, 42) 0 79 28.4 – 29.9 25,105	<0.001
Ratio (food to non-food ads)	1:2.12	1:2.44	

^a^Mann-Whitney U Test to compare differences between school vs non-school groups

Table 2 shows the rate of permitted and non-permitted foods (ads/channel/hour) during school and non-school days. For both periods, there were no observable differences between the rates of non-permitted food advertisements (*p* < 0.001). The rate of non-permitted food advertisements was significantly higher (*p* < 0.001) than for permitted food advertisements during both periods. Almost no permitted food advertisements were observed at any time.

**Table 2.** Rate of children’s exposure to permitted and non-permitted food advertisements (ads/channel/hour) during school and non-school days based on WPRO and SEARO models (α = 0.05).

Indicator (ads/channel/hour)	WPRO Model		SEARO Model	
	Permitted	Non-permitted	Permitted	Non-permitted
School days Mean ± SD Median (IQR) Min Max 95% CI	0.7 ± 1.1 0 (0, 1) 0 7 0.6 – 0.7	9.3 ± 9.7 6 (1, 15) 0 44 8.8 – 9.7	1.0 ± 1.4 0 (0, 2) 0 9 0.9 – 1.0	8.8 ± 9.2 6 (1, 14) 0 42 8.4 – 9.2
*p*^a^	<0.001		<0.001	
Non-school days Mean ± SD Median (IQR) Min Max 95% CI	0.5 ± 0.9 0 (0, 1) 0 5 0.4 – 0.5	8.3 ± 8.5 5 (2, 13) 0 42 8.0 – 8.7	0.5 ± 0.8 0 (0, 1) 0 5 0.4 – 0.5	8.2 ± 8.5 5 (1, 13) 0 42 7.8 – 8.5
*p*^a^	<0.001		<0.001	
*p*^b^	<0.001	0.155	<0.001	0.310

^a^Wilcoxon Signed Ranks Test to compare differences between permitted vs non-permitted food ads

^b^Mann-Whitney U Test to compare differences between school vs non-school groups

Table 3 shows the observed number of permitted and non-permitted food advertisements during peak and non-peak viewing times for children. There were significantly more non-permitted food advertisements compared to permitted advertisements during peak viewing times (*p* < 0.001), and this pattern was the same for school and non-school periods.

**Table 3.** Rate of non-permitted food advertisements (ads/channel/hour) during children’s peak and non-peak viewing times.

Period	WPRO		SEARO	
	PVT	Non-PVT	PVT	Non-PVT
School days Mean 95% CI	11.8 ± 10.6 11.0 – 12.7	8.3 ± 9.2 7.8 – 8.7	11.2 ± 10.0 10.4 – 12.0	7.9 ± 8.7 7.5 – 8.3
Non-school days Mean 95% CI	11.4 ± 9.0 10.6 – 12.1	7.2 ± 8.1 6.8 – 7.6	11.2 ± 8.8 10.5 – 11.9	7.0 ± 8.0 6.6 – 7.4
*p* (PVT vs Non-PVT)	<0.001*		<0.001*	

Mann-Whitney U Test to compare differences between groups.

**Figure 1.**
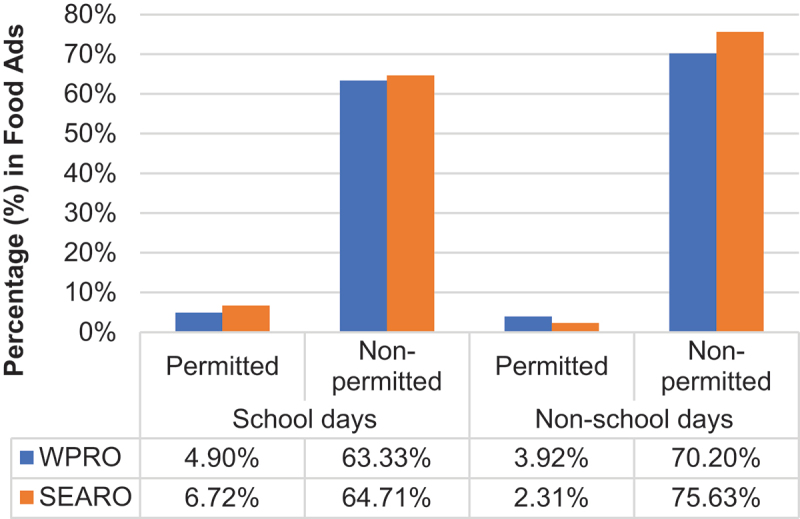
Percentage (%) of permitted and non-permitted food advertisements aired during children’s programs on their peak viewing times.

The study also assessed the percentage of permitted and non-permitted food advertisements observed during children’s programs and their peak viewing times as seen in Figure 1. The proportion of food advertisements that were for non-permitted foods (63–76%) during these times was higher than permitted food advertisements (2–7%). The ratio of permitted to non-permitted food advertisements in children’s programs was 1:13 (WPRO) and 1:10 (SEARO) during school days, and 1:18 (WPRO) and 1:33 (SEARO) during non-school days. Detailed figure comparisons between school and non-school days were also presented in Figure A in the supplementary data.

### Patterns of food advertisements by WPRO and SEARO food categories

**Figure 2.**
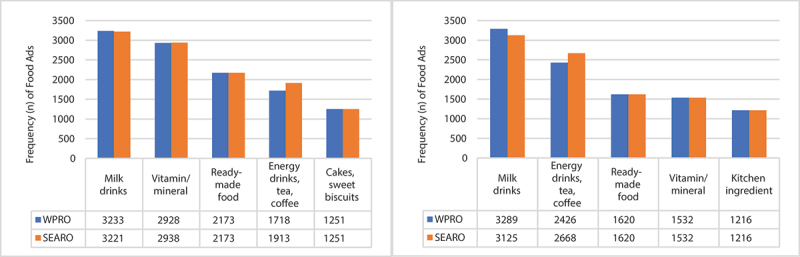
Frequency of top WPRO and SEARO food categories advertised during school days (left) and non-school days (right).

The most frequent WPRO and SEARO food categories of food advertisements aired during school days are (i) milk drinks; (ii) vitamins/minerals; (iii) ready-made food; (iv) energy drinks, tea, coffee (WPRO) or coffee, tea, herbal infusions (SEARO); and (v) cakes, sweet biscuits & pastries. Almost similar patterns are observed during non-school days, except for kitchen ingredients (e.g. seasonings, bouillon cubes, cooking sauces) replacing cakes, sweet biscuits, and pastries in the fifth position. Figure 2 shows a comparison of the frequencies of these food advertisements between WPRO and SEARO models during school and non-school days. Despite milk drinks being the top category in both models, all these food advertisements (100%) were considered non-permitted due to their high added sugar content. On the other hand, vitamins and minerals and kitchen ingredients were classified as ‘not applicable’ under both WPRO and SEARO models. The three remaining categories, namely (i) ready-made food, (ii) energy drinks, tea, and coffee, and (iii) cakes, sweet biscuits, and pastries, are largely considered non-permitted products.

Energy drinks were excluded in the SEARO model (coffee, tea, and herbal infusions only)

To further analyze the content of the advertisements during school and non-school days, Supplementary Figure B shows the frequency of permitted and non-permitted food advertisements based on WPRO and SEARO models during children’s peak viewing time. Most of the aired advertisements are non-permitted foods. The top food categories in WPRO and SEARO models with the most non-permitted advertisements include (i) milk drinks, energy drinks, tea, and coffee (WPRO); or coffee, tea, and herbal infusions (SEARO), (ii) ready-made food, and (iii) cakes, sweets, and pastries.

### Persuasive techniques in food advertisements

Persuasive techniques in food advertisements were classified into two broad categories – powers and premiums. Power strategies referred to marketing techniques are used persuasively to inform the audience about a specific product. Meanwhile, premium strategies referred to the provision of incentives, such as gifts and prizes, to consumers of a product. As seen in Table 4, the top three power strategies observed in food advertisements during school and non-school days were ‘for kids’ (use of child’s image consuming or interacting with the food product, and advertisement lines such as ‘great for school lunches’ or ‘for school lunchboxes’) and the use of celebrity (non-sports). Meanwhile, the top premium strategy observed during the school days was ‘20% extra or other’ (ads with additional product contents). A different pattern was observed during non-school days, where price discounts and game and app downloads were largely observed.

**Table 4.** Frequency analysis [*n* (%)] of top three persuasive techniques used in food advertisements.

Persuasive techniques	School days N (% of all food ads using techniques)	Non-school days N (% of all food ads using techniques)
Power strategies	*n* = 12138	*n* = 9,719
‘For kids’	6895 (56.81%)	5847 (60.16%)
Celebrity (non-sports)	4468 (36.81%)	3213 (33.06%)
Cartoon/company-owned character	324 (2.67%)	340 (3.50%)
Premium strategies	*n* = 717	*n* = 565
‘20% extra’ or other	216 (30.13%)	14 (2.48%)
Game and app downloads	111 (15.48%)	110 (19.47%)
‘Pay 2 take 3’ or other	103 (14.37%)	66 (11.68%)
Limited edition	110 (15.34%)	53 (9.38%)
Price discount	100 (13.95%)	322 (56.99%)

Table 5 shows the frequency analysis of power and premium strategies in non-permitted food advertisements during children’s peak and non-peak viewing times. There were more child-appealing techniques (64–91%) used in non-permitted food advertisements during peak viewing times.

**Table 5.** Power and premium strategies used in non-permitted food advertisements during children’s peak viewing times (PVT) and non-peak viewing times (non-pvt) based on WPRO and SEARO models.

Persuasive strategies	PVT or Non-PVT	WPRO		SEARO	
		Frequency (n)	Percentage (%) of food ads	Frequency (n)	Percentage (%) of food ads
Power strategies					
School days	PVT	2114	64.00	2049	62.03
	Non-PVT	3894	61.39	3803	59.83
Non-school days	PVT	2052	70.71	2093	72.12
	Non-PVT	3404	68.13	3410	68.25
Premium strategies					
School days	PVT	172	75.44	164	71.93
	Non-PVT	309	77.83	295	74.31
Non-school days	PVT	213	91.42	212	90.99
	Non-PVT	286	86.14	278	83.73

To further analyze the use of persuasive strategies, different power and premium strategies in the food advertisements were also analyzed during the peak viewing time of children. For power strategies, the use of kids and celebrities (non-sports) in advertisements was common to almost all food categories (see Supplementary Table A). Premium strategies were commonly observed in advertisements for beverages (see Supplementary Table A). Energy drinks, tea, and coffee products are advertised with ‘20% extra or other’, while other types of sugar-sweetened beverages (e.g. soft drinks, juice drinks) were either advertised with ‘20% extra or other’, game and app downloads, as well as price discounts.

## Discussion

This study aimed to compare Filipino children’s exposure to TV food advertisements during school and non-school days by assessing the level of children’s exposure to food advertisements, examining the healthiness of food advertisements, and identifying the persuasive marketing strategies observed.

It was found that Filipino children are likely to be highly exposed to unhealthy food marketing on television, which could negatively influence their food behavior and food choices. While the rate of food advertisements per hour is significantly lower than that of non-food advertisements, a large percentage of these food advertisements advertise non-core food products that are not permitted under both WPRO and SEARO models. There was a higher rate of food ads per channel per hour during the school days for children (*p* < 0.001). Non-permitted food advertisements were significantly higher (*p* < 0.001) for both time periods during children’s peak viewing times (School: 11.8 ± 10.6 ads/channel/hour, 95% CI 11.0–12.7; Non-school: 11.4 ± 9.0 ads/channel/hour, 95% CI 10.6–12.1) than non-peak viewing times (School: 8.3 ± 9.2 ads/channel/hour, 95% CI 7.8–8.7; Non-school: 7.2 ± 8.1 ads/channel/hour, 95% CI 6.8–7.6). Filipino children are more exposed to non-core food advertisements (7.2–11.8 ads/channel/hour) than other Asian countries like Malaysia (3.8 ads/channel/hour) and China (5.0 ads/channel/hour) [49].

The problem of overweight and obesity can be linked to factors such as poor diets and failing food systems [6], which may all be influenced by exposure to unhealthy TV food marketing among other factors. Excessive exposure to screen time may negatively impact nutrition outcomes, particularly overweight and obesity [32] and unhealthy diet [33]. The results demonstrate the need for food marketing controls to restrict unhealthy food advertising during broadcast periods that attract the greatest child viewers. TV watching in the Philippines is a communal leisure activity in which the program is usually determined by adult family members. For this reason, children are also largely exposed to soap operas and reality TV shows as observed in their peak viewing time schedules from 10:00 to 13:00 and 19:00 to 22:00 hours. Several studies have already emphasized that there is a need to regulate food advertisements even in non-children’s programs as well [50,51].

All programs for school and non-school periods, whether directed toward children or not, advertised foods that were almost exclusively unhealthy. Even without the consideration of being child-directed or not, our assessment of the trends in food advertisements shows that the top food categories being advertised are beverage products such as milk drinks, energy drinks, coffee, and tea. Other top advertised food categories include ready-made food, cakes, sweet biscuits, and vitamins. In our sample, there were no food advertisements for fresh and frozen fruits and vegetables and limited advertisements for processed fruits and vegetables. This trend of high rates of unhealthy food advertisements and low rates of healthy food advertisements is consistent with the results of several related studies [17,52,53].

This study also revealed that the top promotional strategies used in food advertisements were using images of kids and celebrities. Marketing strategies such as these increase children’s food brand recall [54]. Children’s receptiveness to food advertisements varies depending on the age group they belong to, with younger children being more likely to pick advertised items than older children [13]. A study also linked obesity with a child’s responsiveness to food advertisements [55]. Furthermore, parents’ receptivity to food marketing advertisements largely influences children’s diets. Top premium strategies – ‘20% extra’ and others, price discounts, and ‘pay 2 take 3’ or others – are found to target parents more than children. A previous study mentions that parents tend to be receptive to these food advertisements due to various pressures such as lack of time and demands from their children [56]. Our results show that the local Filipino audience may be receptive to price incentives which is why the top premium strategies are those that offer cheaper options. Seasonal premiums are also observed on both school and non-school days. ‘Limited edition’ and ‘game and app downloads’ are one of the top three premiums during school and non-school days. Food brands may offer seasonal premiums to match students’ school years and offer game/app premiums when children are on vacation and more active online. Our study found a significantly higher rate of promotions and premiums used in non-permitted food advertisements, especially during peak viewing times of children. Therefore, these marketing strategies intensify and add to the effects of prolonged exposure to TV food advertisements.

The Philippines lacks a national regulatory mandate for harmful marketing of unhealthy foods and beverages. While the Children’s Television Act of 1997 mandates the monitoring of children’s television programs and advertisements during child-viewing hours [57], and a voluntary, self-regulated pledge – the- “Responsible Advertising to Children Initiative (the Philippine Pledge) [39] also exists, previous studies have shown that self-regulation is ineffective [15,58]. Enforcing stricter advertising guidelines is challenging due to limited regulatory capacity, a lack of stringent, coordinated policies on food and beverage advertising across media platforms, lack of definitive standards in current policies, the lobbying power of the food and beverage industry influencing political will, and weak monitoring and accountability systems during implementation, as evidenced by prior research [59]. The results of this study provide evidence for the large promotion of unhealthy food and drinks on television which are targeted towards children. This highlights the pressing need for regulatory food marketing policies that are in line with global standards to promote children’s health and well-being.

## Strengths and limitations

The study’s findings should be interpreted within the confines of the study’s limitations. First, the assessment of permitted and non-permitted food advertisements was adopted based on the WPRO and SEARO nutrient profile models (NPMs). The models are designed for specific regions (WPRO for Western Pacific and SEARO for Southeast Asia), which have two different sets of specific cut-off values for total fat, saturated fat, total sugars, added sugars, non-sugar sweeteners, sodium, and energy. For instance, the SEARO model does not permit the marketing of products with added sugars. Thus, the two models may have different assessments of the healthiness of advertised foods. Second, both data from school and non-school days were recorded on 16 days randomly selected over three months. More representative and accurate results may be obtained through consistent annual data collection to monitor changes in advertisement trends. Third, the recording period is limited to a three-month duration, and seasonal variations in advertising might have been missed.

Despite the study’s limitations, this study explored exposure levels, healthiness, and persuasive techniques of television food advertisements during school and non-school days for children in the Philippines, which serve as potential source of evidence for policy design improvement on television food marketing regulations in the Philippines. The comparison between school and non-school days captured the distinct patterns in exposure to food marketing across different temporal contexts necessary to develop targeted regulatory policies. The study recorded 18 hours of advertisements in the top three free-to-air channels in the country for each time period. This allowed us to monitor all types of food advertisements observed by children across the whole day, even outside their peak viewing times. This study provides baseline evidence that children’s exposure to television advertising is high which may influence their diet and food choices.

## Policy advocacy actions

The results of this study revealed that higher rates of non-permitted TV food advertisements promoting unhealthy food are observed especially during peak viewing times for children. Thus, this study shows the need for the following policy design and interventions:

1. ***Establishment of TV food marketing regulations to ban the promotion of unhealthy foods and drinks** –* With the dominance of unhealthy food marketing on television, it is necessary to impose strict regulations aimed at restricting and monitoring the promotion of these products to children during peak viewing hours. For example, in countries such as Chile, Canada, and the United Kingdom, mandatory food advertising regulations are imposed to ban the promotion of foods that are high in fat, sugar or salt (HFSS) during media programs with huge child audiences and during specified time periods [60]. Content guidelines should include prohibitions on persuasive marketing techniques (e.g. the use of promotional characters and premium offers) in advertisements for non-permitted foods, as our data showed these strategies were disproportionately used to promote unhealthy food and beverages. Moreover, age-appropriate nutritional information displays should be required, along with robust monitoring and enforcement mechanisms, such as instituting penalties for non-compliance.
2. ***Inclusion of media and nutrition literacy education** –* The Department of Education and Department of Health in the Philippines launched a Healthy Learning Institutions (HLI) framework in basic education schools to strengthen the call for better health and nutrition [61]. This framework could be expanded to include comprehensive media literacy and nutrition education to empower children to consciously make sustainable, healthier food choices and increase their awareness of unhealthy food advertising [62]. Educating children on how to critically assess food advertisements and promoting awareness of healthy nutrition options can foster long-term resilience against harmful marketing influences. Furthermore, nutrition literacy empowers individuals to make informed decisions regarding their eating habits [63].
3. ***Development of child-friendly advertisement standards and inclusion in the comprehensive media program for children*** – There are currently no standards on what foods are considered healthy or not in the television marketing landscape. The National Council for Children’s Television (NCCT) may strengthen and improve its current regulations by integrating TV food marketing restrictions into its current plan to establish a comprehensive media program for children. In the NCCT’s Memorandum Circular No. 2019-01, the agency enumerates its criteria for children’s television programs and child-friendly television programs which includes a section for monitoring the nutrition aspect (Sec. 2a (5): “Nutrition - Portrayals, or references to food and beverages, do not promote or condone unhealthy eating”). This gap shows the need for the agency to redefine this section to aid in the restriction and monitoring of TV food advertisements for children.
4. ***Establishment of clear standards between permitted and non-permitted food advertisements*** – To properly monitor and regulate TV food advertisements, there should be clear standards on what should be considered permitted and non-permitted food products in the Philippines. This can be based on the nutritional content of the advertised food products, WPRO or SEARO models, and persuasive marketing techniques employed.
5. ***Redefinition of Children’s Peak Viewing Times (PVT)*** – Regulation of marketing strategies in food advertisements should consider actual peak viewing times of children which may also include non-children’s programs (e.g. soap operas and reality shows). Current child-viewing hours considered by NCCT are between 8:00 and 11:00 a.m. and/or between 2:00 and 5:00 p.m. However, the results of our study found that 10 a.m. to 1:00 p.m. and 7:00 to 10:00 p.m. have the highest number of children viewers and thus should be considered as peak viewing times.
6. ***Regulation and monitoring of other food marketing media platforms (e.g. social media)*** – Food advertisements are becoming more pervasive on various online media channels apart from television. However, there are no age restrictions on the usage of these platforms unless mediated by parents. The food marketing regulations should have a wider scope to accommodate other platforms that may still influence the food choices of children, particularly towards unhealthy food options.

## Conclusion

This study compared Filipino children’s exposure to TV food advertisements during school and non-school days. Results show that children were potentially more exposed to food advertisements during school days than on non-school days. Based on the WPRO and SEARO nutrient profile models, the rate of non-permitted food advertisements was consistently higher than permitted food advertisements, which implies that children were more exposed to advertisements of non-permitted food advertisements. Filipino children were exposed to a high proportion of advertisements for unhealthy foods during their peak viewing times, with persuasive marketing strategies targeting both children and parents. To address this issue and promote healthier food choices among children, it is recommended that policymakers develop and implement comprehensive child-friendly advertisement standards as part of a broader media program aimed at protecting children’s well-being in the Philippines.

## Supplementary Material

Supplemental Material
