# Supplementary material for: Children’s exposure to unhealthy food advertising on Philippine television: content analysis of marketing strategies and temporal patterns
Source: Glob Health Action. 2024 Nov 21;17(1):2427445. doi: 10.1080/16549716.2024.2427445 (PMC11583323; doi:10.1080/16549716.2024.2427445)
Supplement: Supplemental Material [file ZGHA_A_2427445_SM7347.zip › Supplementary Files/Authors (1).docx]

**Authors’ Bio**

**Elaine Borazon, PhD** is an Assistant Professor at the International Graduate Program of Education and Human Development, College of Social Sciences, National Sun Yat-sen University, Kaohsiung, Taiwan. She holds a bachelor’s degree in food science, and Master’s/PhD degree in Management. Her research interests include food environments, competitive advantage, organizational resilience, and strategy implementation.

**Ma. Rica Sidney S. Magracia** is a graduate student under the International Graduate Program of Education and Human Development, College of Social Sciences, National Sun Yat-sen University, Kaohsiung, Taiwan.

**Gild Rick Ong** is a graduate student at School of BioSciences, Faculty of Health & Medical Sciences, Taylor’s University.

**Bridget Kelly Gillott**, **PhD** focuses on research aiming to influence the development of public policy that fosters supportive food environments in Australia and globally. In particular, her work in critical food marketing seeks to identify the extent that young people are exposed to unhealthy food promotions and the impact that this has on food preferences and consumption behaviours. She is keenly interested in collaborating with international researchers to build and support research in this field at a global level.

**Dr. Sally Mackay**’s career as a public health nutritionist has spanned three decades from working as a health promoter to academic. She began work with the Heart Foundation as a health promoter focusing on caterers and during this time completed a PGDip in Public Health. With a move to Nelson, she worked as a health promoter at the local public health unit and then with the Ministry of Health as senior advisor on the 2008/09 National Nutrition Survey. She completed a PhD at the University of Auckland with the INFORMAS research group on methods to monitor the cost and affordability of diets. This began a research career in monitoring food environments both in Aotearoa and globally with the INFORMAS network. She teaches population health nutrition at the University of Auckland and supervises Masters and PhD students. She is part of research teams developing the methods for the next national nutrition survey and evaluating the National Healthy Food and Drink Policy. Sally recently presented the keynote Muriel Bell lecture at the Nutrition Society of NZ conference in 2022 in recognition of her services to nutrition.

**Boyd Swinburn** is Professor of Population Nutrition and Global Health at the University of Auckland. He trained as an endocrinologist and has conducted research in metabolic, clinical and public health aspects of obesity. His major research interests centre on community and policy actions to prevent childhood and adolescent obesity, and reduce, what he has coined, ‘obesogenic’ environments. He leads the INFORMAS initiative to monitor and benchmark food environments in over 45 countries. He established WHO’s first Collaborating Centre on Obesity Prevention at Deakin University in 2003, led two Lancet Series on Obesity in 2011 and 2015, was co-chair of World Obesity Policy & Prevention section 2009-2019 and co-chair of the Lancet Commission on Obesity 2015-2019. He has been an advisor on many government committees, WHO Consultations, and large scientific studies internationally.

**Tilakavati Karupaiah** is Professor I [Research] at the School of BioSciences, Faculty of Health & Medical Sciences, Taylor's University, Malaysia. Tilaka's research crosses Nutritional Biochemistry, Dietetics, Nephrology and Food Accountability in the Food Environment. Project involvement with Malaysia Lipid Study (MLS), Palm Tocotrienols in Chronic Hemodialysis (PATCH), the Food EPI-BIA Project, IDRC multicountry TV food marketing to children and Rice Interventions for Health (RICH).
