# Supplementary material for: Children’s exposure to unhealthy food advertising on Philippine television: content analysis of marketing strategies and temporal patterns
Source: Glob Health Action. 2024 Nov 21;17(1):2427445. doi: 10.1080/16549716.2024.2427445 (PMC11583323; doi:10.1080/16549716.2024.2427445)
Supplement: Supplemental Material [file ZGHA_A_2427445_SM7347.zip › Supplementary Files/Supplementary Files.pdf]

Appendix A. Supplementary Figures and Tables

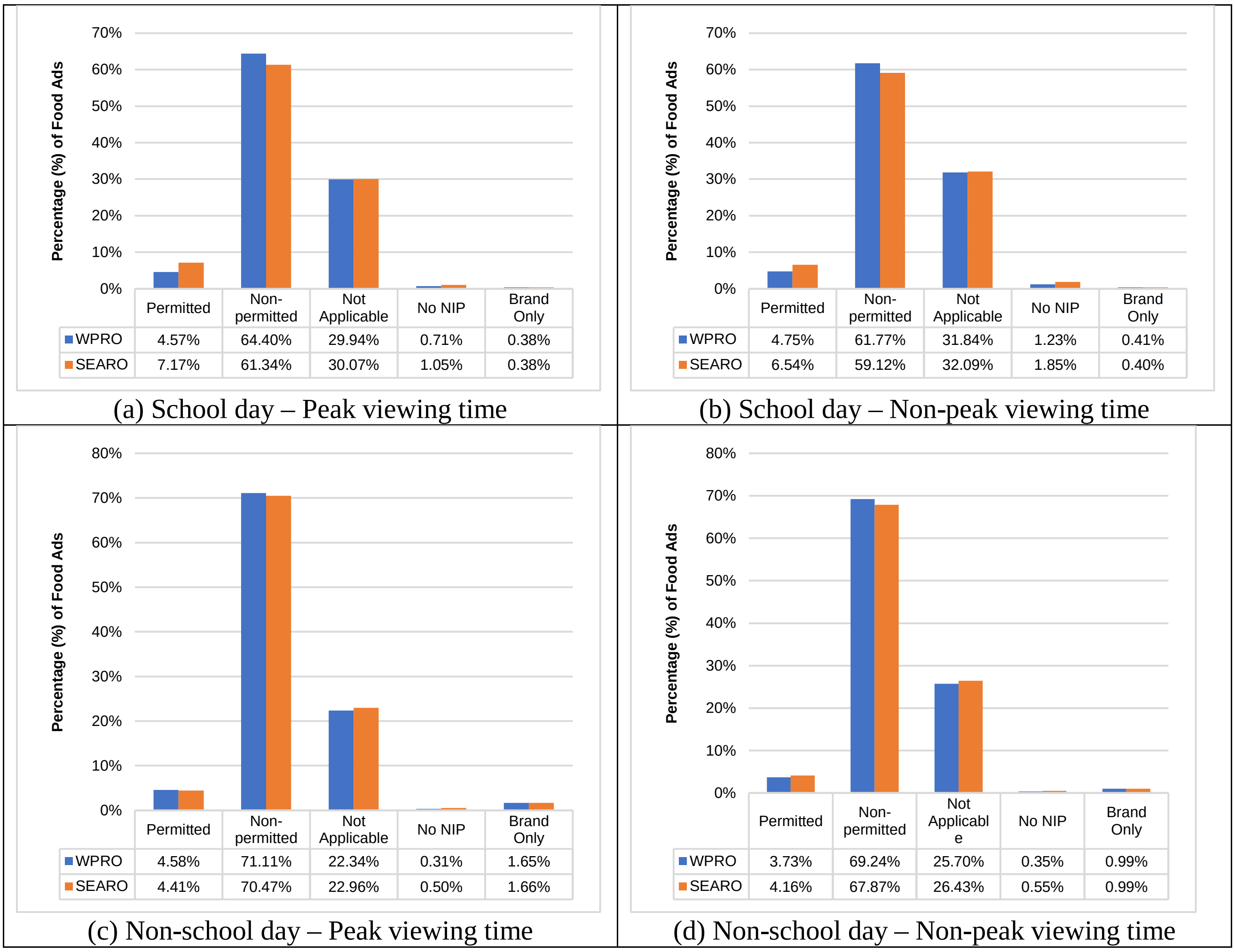

(a) School day – Peak viewing time

(b) School day – Non-peak viewing time

(c) Non-school day – Peak viewing time

(d) Non-school day – Non-peak viewing time

Note: 'Not Applicable'- products in which nutritional information are not-applicable; 'No NIP' – no nutrient information panels; and 'Brand Only' – ads showing company brand only without food products.

Figure A. Percentage (%) of advertisements for permitted and non-permitted foods during children's peak and non-peak viewing times

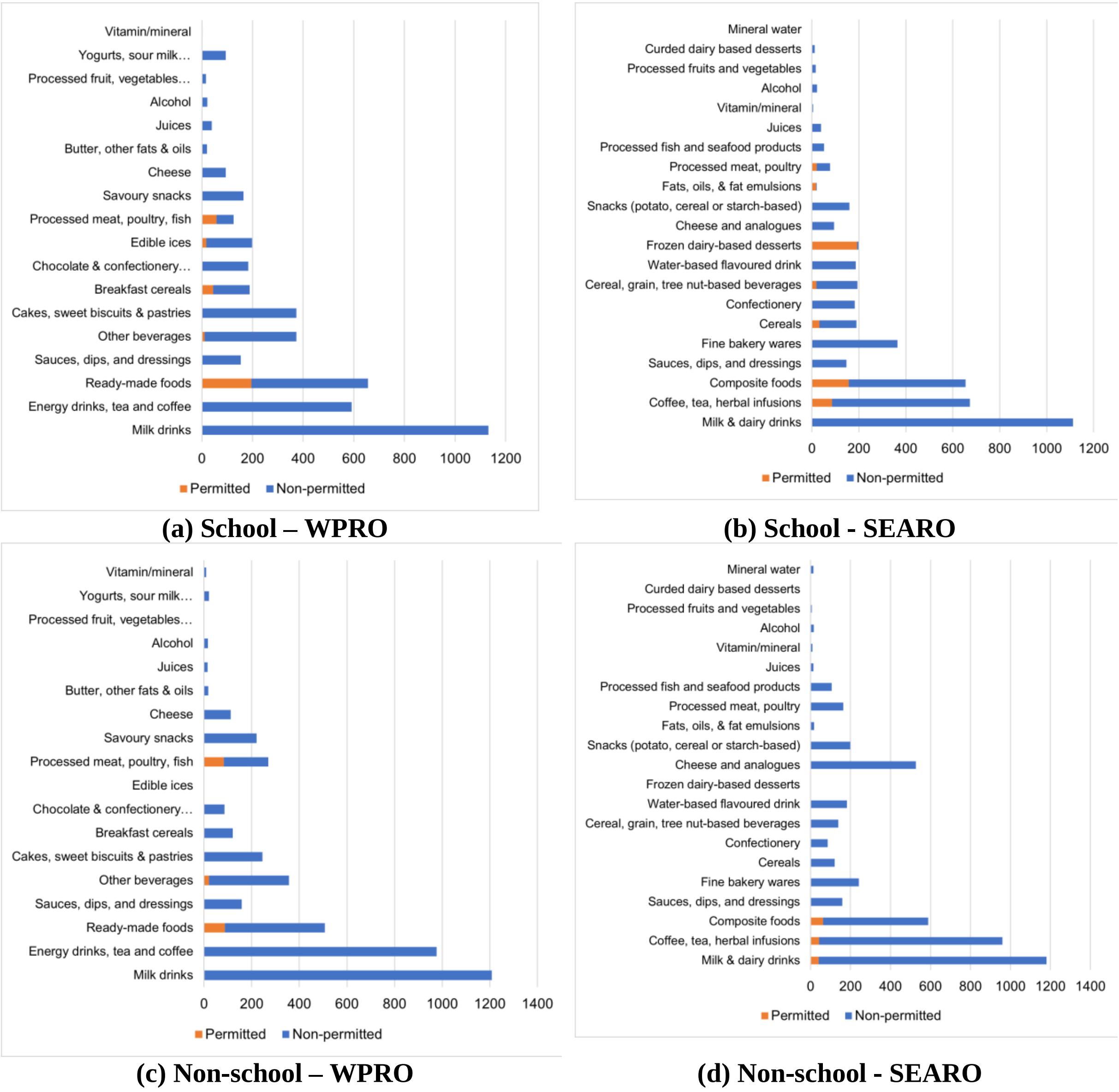

(a) School – WPRO **(b) School - SEARO** **(c) Non-school – WPRO** **(d) Non-school - SEARO**

***Blue bars indicate the large dominance of non-permitted food products**

Figure B. Permitted and non-permitted food advertisements based on WPRO and SEARO models during children's peak viewing time – *(a-b) school; (c-d) non-school.*

Table A. Power and premium strategies observed during peak viewing times of children on school and non-school days

School days

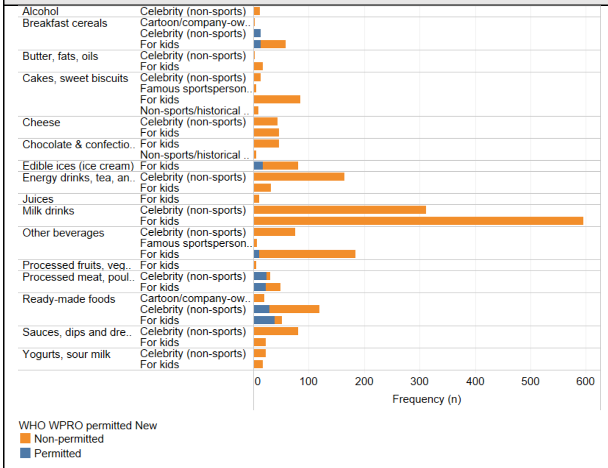

a. Power strategies based on the WPRO model (School)

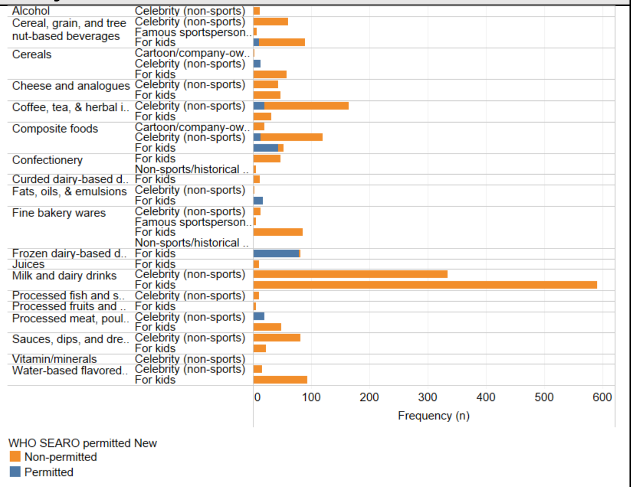

b. Power strategies based on the SEARO model (School)

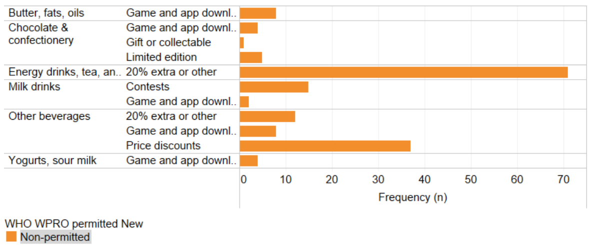

c. Premium strategies based on the WPRO model (School)

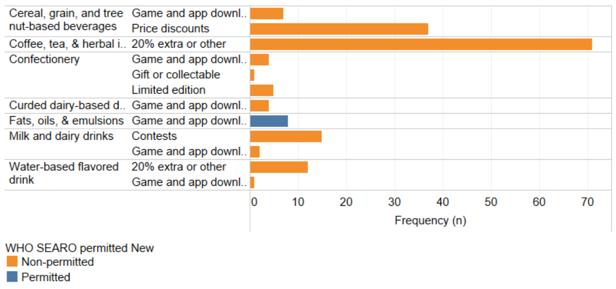

d. Premium strategies based on the SEARO model (School)

Non-school days

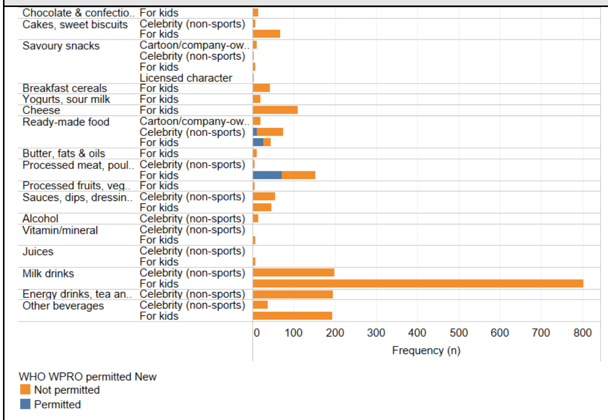

e. Power strategies based on the WPRO model (Non-school)

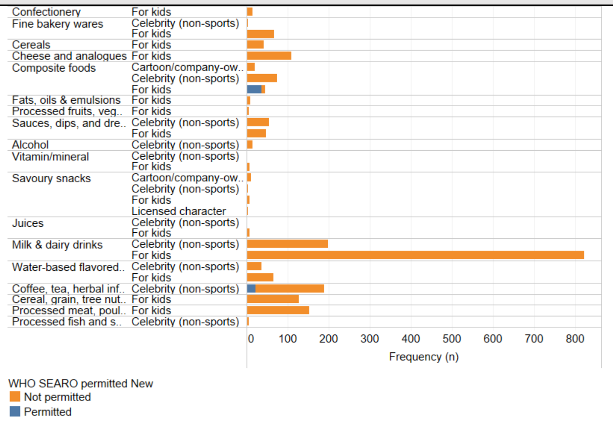

f. Power strategies based on the SEARO model (Non-school)

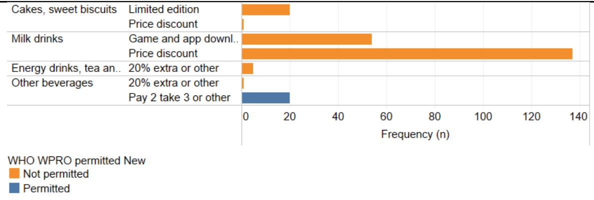

g. Premium strategies based on the WPRO model (Non-school)

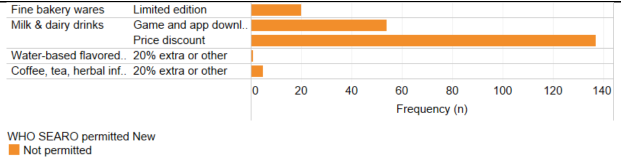

h. Premium strategies based on the SEARO model (Non-school)

Table B. List of Kitchen Ingredients

No.	Kitchen Ingredients
1	Seasoning granules
2	Cooking sauces
3	Recipe premixes
4	Breading mixes
5	Bouillon cubes
6	Pasta sauces
7	Chicken- or beef-flavored evaporated milks
8	Herbs and spices

**Kitchen ingredients were excluded in the analysis of permitted vs. non-permitted food categories*
